# The effect of ketamine on synaptic mistuning induced by impaired glutamate reuptake

**DOI:** 10.1038/s41386-023-01617-0

**Published:** 2023-06-10

**Authors:** Erika Vazquez-Juarez, Ipsit Srivastava, Maria Lindskog

**Affiliations:** 1 Department of Neurobiology, Care Sciences and Society, Karolinska Institutet, 171 77 Stockholm, Sweden; 2 Department of Medical Cell Biology, Uppsala University, 751 24 Uppsala, Sweden

**Keywords:** Synaptic plasticity, Depression

## Abstract

Mistuning of synaptic transmission has been proposed to underlie many psychiatric disorders, with decreased reuptake of the excitatory neurotransmitter glutamate as one contributing factor. Synaptic tuning occurs through several diverging and converging forms of plasticity. By recording evoked field postsynaptic potentials in the CA1 area in hippocampal slices, we found that inhibiting glutamate transporters using DL-TBOA causes retuning of synaptic transmission, resulting in a new steady state with reduced synaptic strength and a lower threshold for inducing long-term synaptic potentiation (LTP). Moreover, a similar reduced threshold for LTP was observed in a rat model of depression with decreased levels of glutamate transporters. Most importantly, we found that the antidepressant ketamine counteracts the effects of increased glutamate on the various steps involved in synaptic retuning. We, therefore, propose that ketamine’s mechanism of action as an antidepressant is to restore adequate synaptic tuning.

## Introduction

A growing body of evidence associates impaired synaptic function with different mental disorders [1, 2]. A plethora of aspects of glutamate transmission and plasticity have lately been suggested to be affected in diseases that targets the brain, including enhanced glutamate transmission, a change in excitation/inhibition balance [3], synaptic plasticity [4] or a change in the homeostatic set-point [2]. Further support for the relevance of excitatory synapse plasticity in psychiatric disorders in general—and depression in particular—comes from the finding that subanesthetic doses of the *N*-methyl-D-aspartate (NMDA) receptor antagonist ketamine produce a fast-acting antidepressant effect [5, 6]. Although our understanding of ketamine’s mechanism of action is still ambiguous [7, 8], ketamine has been shown to beneficially affect glutamatergic transmission [9, 10]. The therapeutic effects were originally attributed to a reduction in excitatory activity by inhibiting NMDA receptors; however, further research revealed that ketamine can have complex effects on the balance between excitation and inhibition and on synaptic plasticity [7, 11, 12], including——paradoxically—an increase in excitatory transmission through decreased inhibition and/or synaptic plasticity mechanisms [13].

The role of synaptic deficits in disease is a reflection of the need of a well-tuned and balanced excitatory synaptic transmission to ensure healthy brain function. The balance is achieved through interactions of various forms of plasticity. For example, in Hebbian (i.e., activity-dependent) plasticity, coordinated neuronal activity generates a positive feedback process that reinforces the strength of specific synapses with high levels of activity [14]. Conversely, homeostatic plasticity uses negative feedback to downregulate synaptic activity in response to high levels of global activity [15]. The interplay between different forms of plasticity, including their effect on each other, known as metaplasticity, is fundamental for ensuring balanced neuronal activity while providing the flexibility needed to maintain brain function [see, for example [16, 17]. However, our knowledge regarding the interaction between various forms of plasticity is based to a large extent on indirect measurements such as changes in receptor expression and synapse morphology [18, 19].

Interestingly, one key mechanism for keeping excitatory neurotransmission balanced - namely glutamate reuptake - appears to be affected in many mental disorders. In particular, the astrocytic glutamate transporters EAAT-1 and EAAT-2, which take up the majority of synaptic glutamate [20], have consistently been reported to be expressed at reduced levels in individuals with cognitive and/or psychiatric disorders [21, 22]. Increased levels of extracellular glutamate are typically believed to lead to excitotoxicity; however, reduced glutamate reuptake can induce a number of adaptive changes and does not necessarily induce cell death [23, 24]. To date, strikingly little is known regarding the process by which synaptic tuning is affected when glutamate transporter levels are reduced.

Considering the consistent finding of downregulated glutamate transporters in animal models of depression as well as in patients with depression, and given the association between synaptic plasticity and depression, it is of great relevance to understand how the tuning of excitatory synapses is affected by reduced glutamate reuptake. We, therefore, measured evoked synaptic responses in rat hippocampal slices while blocking glutamate transporters and found that a series of retuning steps lead to a new steady state in which the threshold for inducing plasticity is reduced. Moreover, we found that ketamine was effective at reversing all of these retuning steps, providing new insights into its mechanism of action as an antidepressant drug.

## Material and methods

### Drugs

DL-TBOA, DL-AP-5 sodium salt, MK-801 maleate, ketamine hydrochloride, NBQX disodium salt, were purchased from Tocris Bioscience (Bristol, UK).

### Animals

All experiments were approved by the regional committee for animal research of Stockholm North in Sweden (N13/15). Unless stated otherwise, male adult (6–9 weeks of age) rats were used. Sprague-Dawley (SD) rats were supplied from Janvier Laboratories, and FSL rats were bred at the Karolinska Institute. All rats were group-housed at a 12-h light/dark cycle in the animal facility at Karolinska Institute and had access to food and water *ad libitum*. For slice preparation, the rats were deeply anesthetized with isoflurane and decapitated shortly after loss of the corneal reflex

### Hippocampal slice preparation

Immediately after decapitation, the brain was dissected and placed in ice-cold artificial cerebrospinal fluid (aCSF) containing (in mM): 124 NaCl, 30 NaHCO_3_, 10 glucose, 1.25 NaH_2_PO_4_, 3.5 KCl, 1 MgCl_2_, and 2 CaCl_2_. Horizontal hippocampal slices (400-µm thickness) were prepared using a Leica VT1200 vibratome (Leica; Deerfield, IL) and placed in an interface incubation chamber containing aCSF. The chamber was kept at 34 °C during slicing and then returned to ambient room temperature for at least 2 h. The slices were continuously bathed in aCSF bubbled with humidified carbogen gas (5% CO_2_/95% O_2_).

### fEPSP recordings

Acute hippocampal slices were transferred to a submerged recording chamber and continuously perfused at 2–3 ml/min with aCSF at 32 °C. An Ag/AgCl extracellular electrode with a borosilicate pipette filled with aCSF was placed in the stratum radiatum, and field excitatory postsynaptic potentials (fEPSPs) were evoked by electrical stimulation of the Schaffer collaterals (50-µs duration) using a bipolar concentric electrode (FHC Inc., Bowdoin, ME) connected to an isolated current stimulator (Digitimer Ltd., Welwyn Garden City, UK). Recordings were performed with the stimulus intensity set to elicit 30–40% of the maximal response (typically 30–50 µA), and individual synaptic responses were evoked at 0.1 Hz (every 10 s), unless otherwise stated. For recording the NMDA receptor‒mediated component of the fEPSP, responses were evoked in the presence of 50 µM NBQX with 0.2 mM MgCl_2_ in the aCSF; at the end of the recording, the specificity of the signal was confirmed by applying 25 µM AP5. The acquired signal was amplified and filtered at 2 kHz (low-pass filter) using an extracellular amplifier (EXT-02F, NPI Electronic, Tamm, Germany). Data were collected and analyzed using a Digidata 1440 A, Axoscope, and Clampfit (Molecular Devices, Can Jose, CA). Responses were quantified by determining the slope of the linear rising phase of the fEPSP (between 10% and 70% of the peak amplitude). The response was then normalized to the average baseline measured in the last 5 min prior to the start of the experiment. Time courses were created by averaging 6 fEPSPs collected during 1 min. For fEPSP responses evoked at 0.001 Hz, 3 single stimuli delivered at 0.1 Hz were applied and averaged at 15-min intervals. To induce LTP in the CA1 region, theta-burst stimulation (θ-burst; 10 bursts of 4 pulses at 100 Hz, delivered at 5 Hz) was applied twice with a 10-s interval. For subthreshold stimulation, the number of θ-bursts was determined in control slices at the beginning of each experiment and ranged from 5 to 8 bursts. Where indicated, the data were re-normalized to the average of the last 5 min prior to inducing LTP. The presence of LTP was determined by comparing the average of the fEPSP slope measured 55–60 min after θ-burst stimulation with the average of the last 5 min prior to stimulation. For group comparisons, time-matched 5-min averages were used, unless otherwise indicated.

### Patch-clamp recordings

After the acutely prepared slices recovered for at least 1 h, they were transferred to a submerged recording chamber perfused at 2–3 ml/min with aCSF at 32 ± 1 °C. The Schaffer collaterals were stimulated at a frequency of 0.1 Hz using a bipolar electrode, and the corresponding field potential was recorded. After a baseline recording of 10 min, DL-TBOA (50 μM) was added. During 40 min in DL-TBOA, the synaptic retuning and new steady state were induced and confirmed as above, after which pyramidal neurons in the CA1 area (identified by their shape and localization in the pyramidal cell layer) proximal to the recording electrode were recorded in patch-clamp experiments using a Ag/AgCl electrode in a borosilicate glass pipette with a tip resistance of 4–5 MΩ filled with a solution containing (in mM): 110 K-gluconate, 10 KCl, 4 Mg-ATP, 10 Na_2_-phosphocreatine, 0.3 Na-GTP, 10 HEPES, and 0.2 EGTA (pH 7.2–7.4; 270–290 mOsm). To isolate miniature EPSCs arising from spontaneously released synaptic vesicles, 1 µM tetrodotoxin was added to the perfusion solution to block action potentials. Access resistance was monitored throughout the recordings, and only stable recordings (<20% variation) were included in our analysis. Data were acquired using a Multiclamp 700B amplifier and Clampex 10.0 (Molecular Devices) and digitized using a Digidata 1440 A (Molecular Devices). Traces were analyzed using the Mini Analysis Program (Synaptosoft Inc., Fort Lee, NJ).

### Enzyme-based glutamate sensor

The ceramic-based microelectrode array (MEA; CenMeT Service Center, Lexington, KY) consisted of two 50 × 150 μm platinum recording sites arranged in a row with 50 μm spacing between sites. One recording site was designed to be sensitive to glutamate and was coated with 2% glutamate oxidase (US Biological, G4001-01), 1% BSA (Sigma-Aldrich, A3059), and 0.125% glutaraldehyde (Sigma-Aldrich, G5882); the other site served as a sentinel detector and was coated only with BSA and glutaraldehyde. To prevent potential interference from reaching the MEA surface and to increase selectivity, the enzyme-coated MEAs were electroplated with the size-exclusion polymer m-phenylenediamine (mPD) by cycling between +0.2 V and +0.8 V at 50 mV/s for 22 min in a nitrogen-bubbled solution containing 10 mM mPD. Coated MEAs were allowed to dry for 48 h at room temperature in low humidity prior to in vitro calibration. The glutamate signal was obtained by subtracting the signal measured at the sentinel site from the signal measured at the enzyme-coated site.

The microelectrodes were calibrated each day prior use in slice recording. Constant potential amperometry was performed by applying a potential of +0.7 V relative to an Ag/AgCl reference electrode. Calibrations were performed in a stirred solution of 0.05 M phosphate-buffered saline (pH 7.4, 37 °C). After a stable baseline signal was achieved, 250 µM ascorbic acid, 20 µM L-glutamate (3x), 2 µM dopamine (freshly prepared), and 8.8 µM H_2_O_2_ were added sequentially to the calibration beaker. Amperometric signals were acquired at 0.1 Hz using a FAST-16 mk-I electrochemical recording system (Quanteon LLC, Nicholasville, KY) and analyzed off-line using FAST Analysis software (Quanteon LLC).

### Propidium iodine (PI) exclusion

To measure cell viability, slices were prepared as described above. After recovering in the interface chamber, the slices were transferred to a submerged chamber and incubated with 50 µM DL-TBOA or 10 mM NaN_3_ for 1 h. The slices were then transferred to an incubation chamber containing propidium iodine (1 µg/ml) for 15 min, washed, and mounted in medium containing DAPI (Vector Laboratories, Burlingame, CA). The slices were imaged using an epifluorescence microscope, and both PI-positive cells and DAPI-stained nuclei were counted. The fraction of dead cells was calculated as the number of PI-positive cells divided by the number of DAPI-stained nuclei.

### Data analysis and statistics

Results are shown as averages of each recording (slice), and at least 3 animals were used per condition unless otherwise noted. For statistical analysis, each recording is an independent variable. Experimental groups were compared with Student *t*-test in the case of two groups, or for multiple groups ANOVA, followed by post-hoc test. All statistical analysis was done using Prism software (Graph Pad, San Diego, CA, USA).

## Results

### Blocking glutamate transporters induces synaptic retuning and increased levels of extrasynaptic glutamate through activation of NMDA receptors

**Fig. 1:**
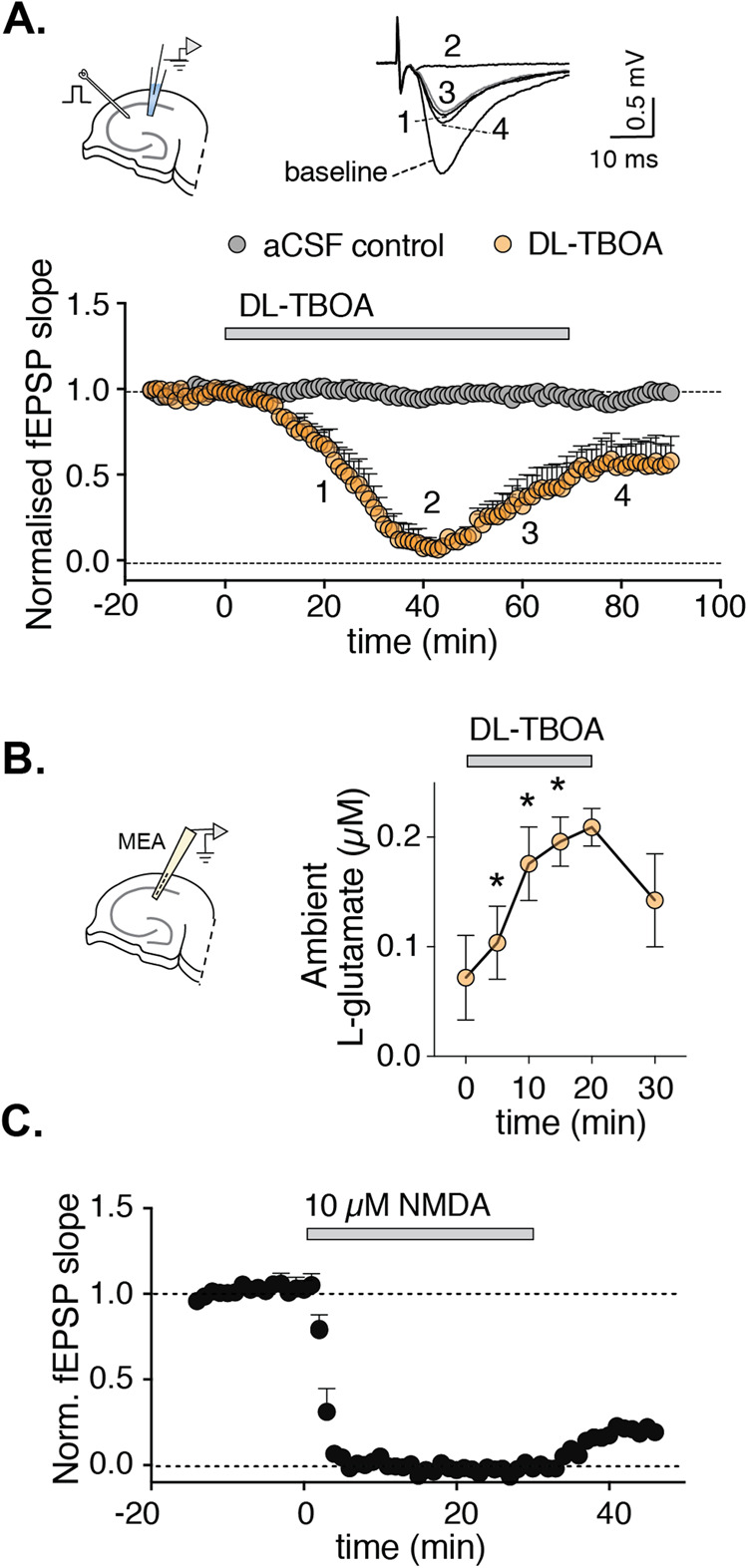
Increasing extrasynaptic glutamate by blocking glutamate transporters induces synaptic retuning. **A** Blocking glutamate transporters induces a dynamic change over time of fEPSP magnitude in rat hippocampal slices, shown as normalized fEPSP slope. The Schaffer collaterals were stimulated at 0.1 Hz throughout the experiment while recording from the stratum radiatum at the CA1 area (scheme top left). The fEPSP slope over time is plotted as average of 6 stimulation pulses (corresponding to 1 min) in this and subsequent figures. Where indicated by bar, the glutamate transporter inhibitor DL-TBOA (50 µM) was applied and then washed out (bottom, *n* = 4 and 5 slices for aCSF control and DL-TBOA, respectively). One-minute averaged example traces (top right) of fEPSP recorded at baseline and at indicated time-points (20, 40, 60 and 80 min after DL-TBOA application). **B** Extracellular glutamate recordings in the CA1 using enzyme coated microelectrodes (scheme left) show an average increase of basal level of extracellular glutamate immediately upon application of 50 µM DL-TBOA (right; *n* = 8 slices). **C** Synaptic retuning can be mimicked by NMDA. Time course of normalized fEPSP slope where 10 µM NMDA was added as indicated by bar (*n* = 4 slices). In this and subsequent figures, data is represented as average of each recording, and error bars denote the standard error of the mean (SEM). *p < 0.05 in *t*-test or ANOVA followed by post-hoc test if more than two groups are compared.

The acute effect on reduced glutamate transporter function was examined by recording field excitatory postsynaptic potentials (fEPSPs) in the CA1 area in response to presynaptic activation of the Schaffer collaterals. Bath application of the glutamate transporter inhibitor DL-threo-beta-benzyloxyaspartate (DL-TBOA) caused a gradual but transient decrease in the magnitude of the synaptic response, quantified by the slope of the rising phase of the evoked fEPSPs (see Methods; Fig. 1A). A persistent disruption of glutamate reuptake, by the presence of DL-TBOA induced the complete loss of the synaptic response within 40 min (*p* < 0.001 compared to aCSF control at 35–45 min) that was followed by a recovery of the response to 44% of baseline, that is significantly different from aCSF treated slices (*p* < 0.01 compared to mock treated slices at 60–70 min) (Fig. 1A). In contrast, DL-TBOA did not affect fiber volley amplitude (Supplementary Fig. S1), indicating that the stimulating input was unchanged. Washing out DL-TBOA lead to a larger recovery of fEPSP magnitude, albeit to only 56% of baseline on average and still significantly different from mock-treated slices (*p* < 0.01, *t*-test at 10–20 min after wash-out). Thus, blocking glutamate reuptake induces several steps of dynamic adaptation or retuning of synaptic transmission, reaching a new steady state in about 1 h. Similar results were obtained using TFB-TBOA, another glutamate transporter inhibitor with high specificity for transporters expressed in astrocytes. (Supplementary Fig. S1); However, because the effects of TFB-TBOA on the synaptic response were more variable, DL-TBOA was used for subsequent experiments. We ruled out a toxic effect of DL-TBOA in the slices by propidium iodide uptake experiments (a measure of cell death), finding no significant difference between DL-TBOA‒treated slices and control-treated slices (Supplementary Fig. S1). Likewise, when spontaneous synaptic events were recorded in the presence of tetrodotoxin in patch-clamped pyramidal neurons, we found no significant difference in the frequency of mEPSCs between slices that had been retuned with DL-TBOA compared to control slices (Supplementary Fig. S2), ruling out a loss of synapses.

To confirm that DL-TBOA did indeed induce an increase in extracellular glutamate, direct recordings of the extracellular level of glutamate using enzyme-based amperometric glutamate sensors were performed. Continuous application of DL-TBOA for 20 min significantly increased the basal level of extracellular glutamate from average of 72 + /− 38 nM to 196 nM +/− 55 (*p* < 0.05, ANOVA, followed by Dunetts multiple comparison test; Fig. 1B). The extracellular glutamate level after evoked transmission however, was not significantly different in the presence of DL-TBOA (Supplementary Fig. 2). Interestingly, the increase in glutamate level occurred in a faster time scale than the modifications observed in the fEPSP, significantly increased level of extracellular glutamate were detected within 5 min (104 nM +/− 33 (*p* < 0.05, ANOVA, followed by Dunetts multiple comparison test) and reached a steady-state after 10 to 20 min.

Consistent with increased extracellular glutamate causing retuning of synaptic transmission, direct application of glutamate at 1 mM, but not 100 μM, induced a similar response as DL-TBOA (Supplementary Fig. 3). Moreover, sustained application of NMDA (10 µM NMDA, 30 min) to stimulate glutamate NMDA receptors, partially mimics the effect of DL-TBOA, inducing a decrease in the magnitude of evoked fEPSPs that remained after wash-out (*p* < 0.001 baseline compared to 40–45 min after application of NMDA). The response to NMDA is, however, faster than the response induced by DL-TBOA and the fEPSP reaches a minimum within 4 min, compared to 35 min for DL-TBOA treated slices (Fig. 1C), and the recovery of the fEPSP is only observed after wash-out The difference in response to the two drugs could be due to the different kinetics of glutamate receptor activation and recruitment induced by a gradual increase in glutamate levels after the application of DL-TBOA compared to the sudden activation of NMDA receptors by an acute application of 10 µM NMDA. Taken together our results show that blocking glutamate transporters with DL-TBOA induces a fast increase in extracellular glutamate that can activate glutamate receptors, including NMDA receptors, followed by a retuning of synaptic transmission.

### The threshold for synaptic potentiation is reduced at the new steady state after synaptic retuning

**Fig. 2:**
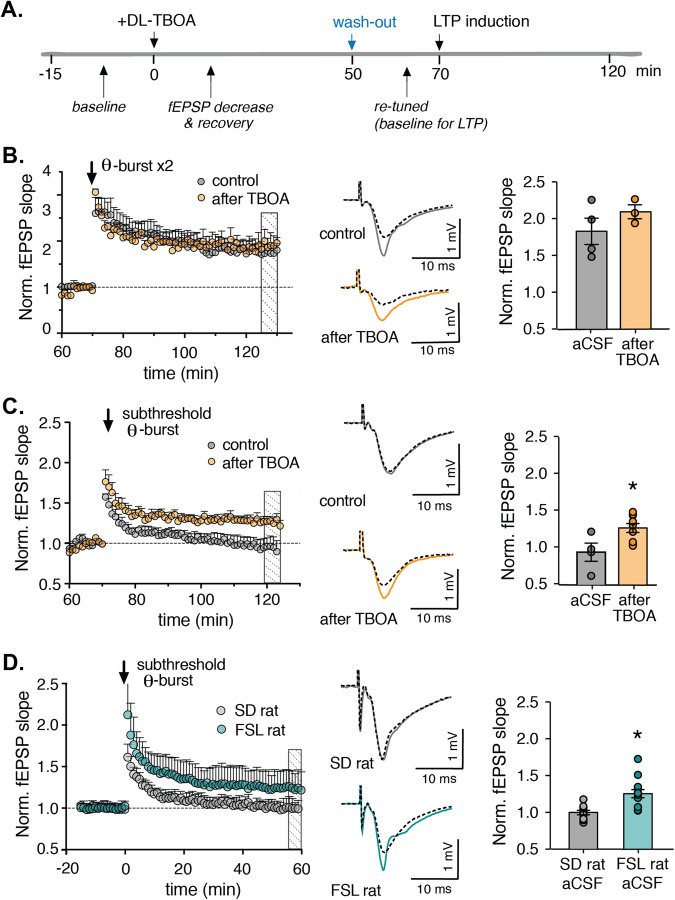
Decreasing glutamate uptake lowers the threshold for inducing long-term potentiation. **A** Protocol used to induce a new steady state in hippocampal slices by applying 50 µM DL-TBOA for 40 min while stimulating the Schaffer collaterals. Control slices are treated similarly but are perfused with aCSF instead of DL-TBOA. At 70 min, when the new steady state is reached, a theta-burst (θ-burst) stimulation protocol is applied. **B** Time course of normalized fEPSP slope in control slices (*n* = 4) and retuned slices (*n* = 3); where indicated, a standard LTP-inducing θ-burst was applied. Representative traces shown at right. Average fEPSP magnitude compared to baseline at 55–60 min after θ-burst shown to the right. **C** Time course of normalized fEPSP slope in control slices (*n* = 4) and retuned slices (*n* = 10); where indicated, a subthreshold θ-burst stimulation was applied. Representative traces shown at right. Average fEPSP magnitude compared to baseline at 55–60 min after θ-burst to the right, showing a significant difference in DL-TBOA treated versus mock-treated slices. **D** A subthreshold θ-burst stimulation was applied in slices obtained from either Sprague-Dawley (SD) rats or FSL rats. Time course of normalized fEPSP slope in SD (*n* = 10) and FSL rats (*n* = 13); where indicated, a standard LTP-inducing θ-burst was applied. Representative traces shown at right. Histogram to the right shows average fEPSP magnitude compared to baseline at 55–60 min after θ-burst, showing a significant difference in FSL versus SD rats.

The finding that blocking glutamate reuptake for a prolonged period, triggers a dynamic response that retunes the synapse, prompted us to investigate the synaptic plasticity at the new steady state. Plasticity was measured in slices at the new steady state, where DL-TBOA had been applied and then washed out, to eliminate the direct effect of high level of glutamate rather than the long-lasting modifications in synaptic transmission that had been established during retuning (Fig. 2A). When long-term potentiation (LTP) was induced with two trains of 10 stimuli at 5 Hz (i.e., θ-burst stimuli) there was no difference in LTP between DL-TBOA‒treated slices and control slices (*p* = 0.29 at 55–60 min after θ-burst stimulation; Fig. 2B). To our surprise, however, we found that a subthreshold θ-burst stimulation elicited only short-term potentiation in control slices (*p* = 0.99 compared to baseline at 50–55 min after stimulation, ANOVA followed by Sidak post-hoc comparison) but induced LTP in slices that had been retuned with DL-TBOA (*p* < 0.001 compared to baseline at 50–55 min after stimulation, ANOVA followed by Sidak post-hoc comparison; Fig. 2C, full timeline in Supplementary Fig. S4). Thus, the new steady state induced by increasing extracellular glutamate renders the synapses more susceptible to long-term plasticity.

To investigate the translational relevance of the reduced threshold of plasticity, we examined the effect of subthreshold stimulation in hippocampal slices prepared from FSL (Flinders Sensitive Line) rats, an established model of depression in which glial glutamate transporters are downregulated [23, 25]. We found that the same subthreshold θ-burst stimulation that did not induce LTP in slices prepared from control (Sprague-Dawley, or SD) rats (*p* > 0.99 baseline versus 55–60 min after stimulation, ANOVA followed by Sidak post-hoc comparison), was sufficient to induce robust LTP in slices prepared from FSL rats (*p* < 0.01, baseline versus 55–60 min after stimulation, ANOVA followed by Sidak post-hoc comparison; Fig. 2D), similar to what we observed after DL-TBOA treatment.

### The recovery of fEPSP magnitude is dependent on presynaptic activity

**Fig. 3:**
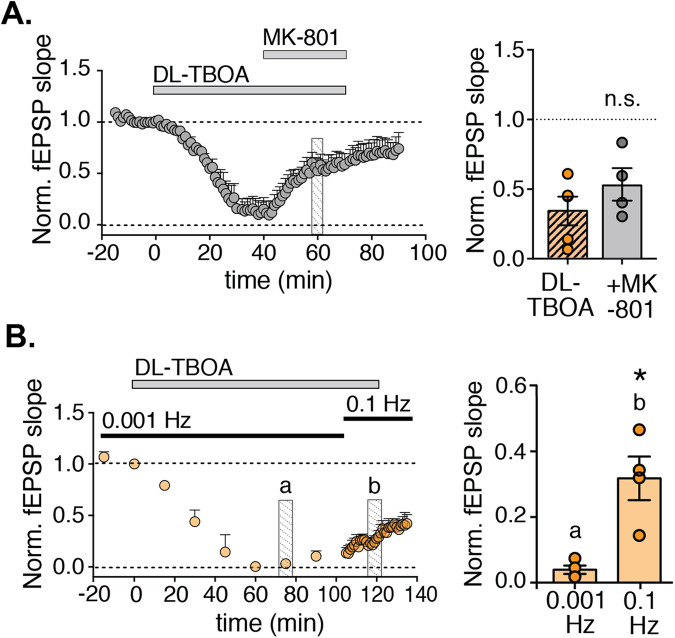
The recovery of fEPSPs does not require NMDA receptor activity but is dependent on stimulation frequency. **A** Time course of normalized fEPSP slope; where indicated DL-TBOA and MK-801 were added (*n* = 4 slices). Histogram to the right shows average fEPSP magnitude 20–25 min after MK-801application compared to the same time in DL-TBOA only treated slices (same data as in Fig. 1A, showing no statistical difference. **B** Time course of normalized fEPSP slope measured using the indicated stimulation frequencies; where indicated, DL-TBOA was added (*n* = 5 slices). Right: Summary of average normalized fEPSP slope measured at the indicated times in **B**. *p < 0.05 compared to 0.001 Hz (Mann–Whitney *U* test).

Despite the fact that NMDA receptor activation could partially mimic the retuning induced by DL-TBOA treatment, the recovery of the evoked response during DL-TBOA treatment and the establishment of the new steady state (labeled 3 in Fig. 1A) did not require NMDA receptor activation: the addition of MK-801 when the fEPSP magnitude had reached its minimum, did not prevent the recovery (Fig. 3A). If anything, the new steady state was reached faster in the presence of MK-801. In contrast, the recovery - but not the initial decrease - was dependent on the frequency of the stimulation used to evoke field responses (Fig. 3B). When fEPSPs were evoked every 15 min (i.e., at approximately 0.001 Hz) during DL-TBOA treatment, the initial decrease of fEPSP magnitude was observed, however, no recovery of the response occurred. When the stimulation frequency was then switched to 0.1 Hz, however, the fEPSP response recovered (*p* < 0.05 before versus 10 min after increasing the stimulation frequency, Mann–Whitney test), indicating that recovery from the non-responding state in the presence of DL-TBOA requires presynaptic activation (Fig. 3B). This result highlights the complexity of the dynamic response triggered by DL-TBOA treatment and the role of multiple players in the establishment of the new steady state.

### The retuning of synaptic response is dependent on NMDA receptor activation

**Fig. 4:**
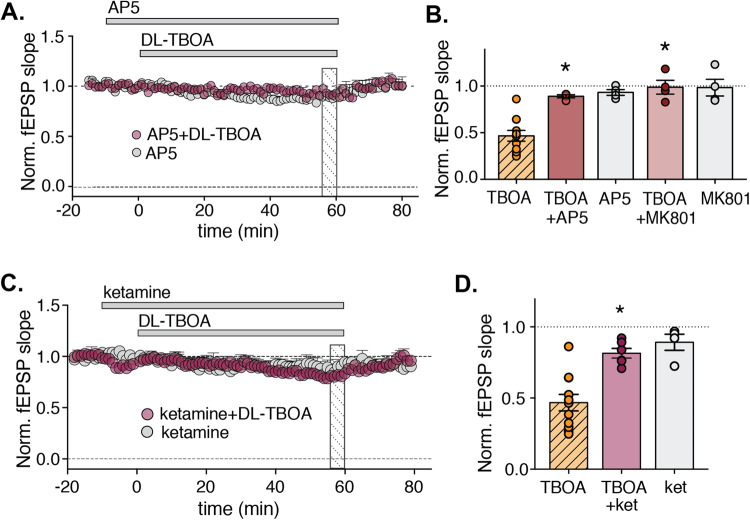
The decrease in fEPSP magnitude induced by DL-TBOA requires NMDA receptor activity. **A** Adding 10 µM AP5 in the absence or presence of DL-TBOA does not affect the fEPSP magnitude (*n* = 4/condition). **B** Average normalized fEPSP slope measured 56 to 60 min after application of the indicated compounds, hatched bar indicates that the data has been shown in previous figure. **C** Time course of normalized fEPSP slope in acute slices from SD rats; 20 µM ketamine and/or DL-TBOA were applied as indicated (*n* = 4–6 slices each). **D** Average fEPSP slope normalized to baseline measured 55–60 min after the application of 50 µM DL-TBOA (hatched bar indicates that the data has been shown in previous figure), 20 µM ketamine (*n* = 4 slices), or 20 µM ketamine + 50 µM DL-TBOA (*n* = 6 slices).

In contrast to the recovery and in line with the partial replication of the DL-TBOA effect by NMDA application, the initial decrease in fEPSP magnitude in response to DL-TBOA was NMDA receptor dependent. When slices were pretreated with either the competitive NMDA receptor antagonist AP5 or the NMDA receptor pore blocker MK-801, the DL-TBOA‒induced decrease in fEPSP magnitude was prevented (Fig. 4A, B). Neither drug had any effect on the fEPSP magnitude in the absence of DL-TBOA (Fig. 4A, B). Moreover, we confirmed that DL-TBOA application induced an increase in NMDA receptor mediated synaptic responses that could trigger the retuning. When the fEPSP was recorded in the presence of 25 µM NBQX to block AMPA receptors and 0.2 mM MgCl_2_ to favor NMDA receptor opening, a transient increase in the magnitude of the NMDA receptor-mediated component of the fEPSP was observed upon addition of DL-TBOA (*p* < 0.05 compared to control recordings, Mann–Whitney *U* test; Supplementary Fig. S5).

Having confirmed the relevance of the new steady state in an animal model of depression, and given that the fast-acting antidepressant drug ketamine is an NMDA receptor antagonist, we tested whether a subanaesthetic dose of ketamine, a concentration that blocks about 50% of NMDA receptors [26, 27], can prevent the DL-TBOA‒induced decrease in fEPSP magnitude. Similar to our results obtained with the NMDA receptor blocker MK-801 and the NMDA receptor antagonist AP-5, we found that 20 µM ketamine eliminated the ability of DL-TBOA to decrease fEPSP magnitude in rat hippocampal slices (*p* < 0.01 DL-TBOA versus DL-TBOA+ketamine at 55–60 min after DL-TBOA application, ANOVA followed by Dunnets test; Fig. 4C, D). Ketamine did not have any effect on the fEPSP magnitude in the absence of DL-TBOA (Fig. 4C, D).

### The antidepressant drug ketamine affects the retuning of synaptic transmission

**Fig. 5:**
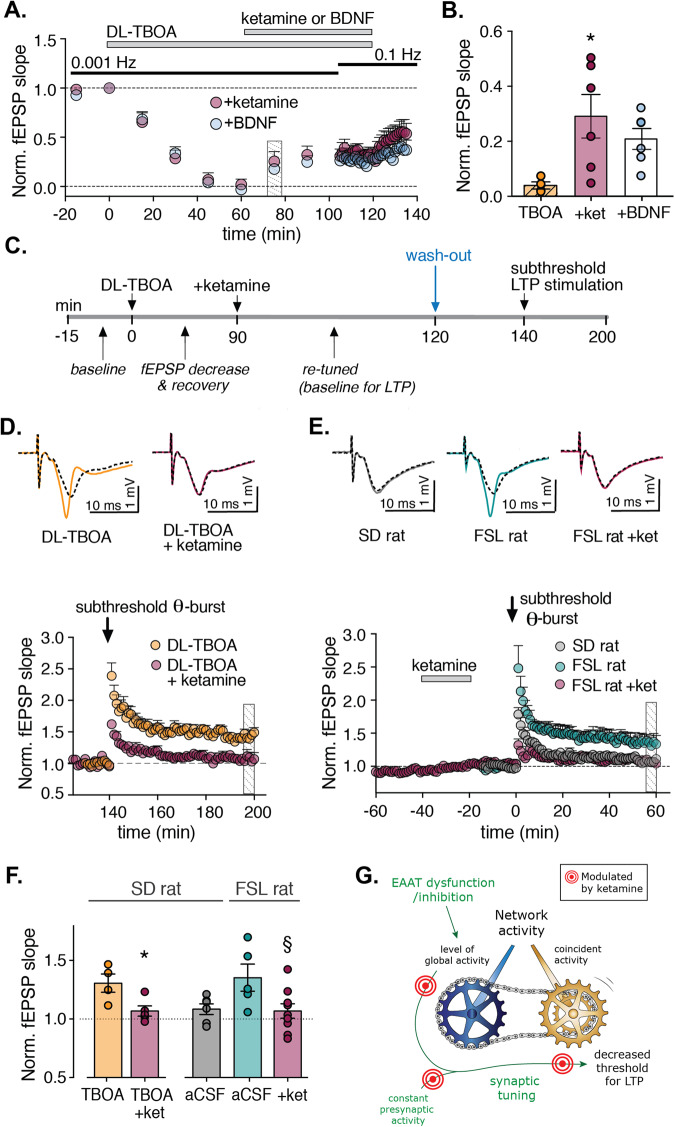
Ketamine affects synaptic tuning at multiple steps. **A** Time course of normalized fEPSP slope while stimulating at the indicated frequencies; where indicated, DL-TBOA and either ketamine or BDNF were applied (*n* = 6 slices each). **B** Average fEPSP slope measured 15 min after ketamine or BDNF (see **A**) versus DL-TBOA alone (from Fig. 4B-a). *p < 0.05 versus DL-TBOA alone (ANOVA). **C** Protocol used to test the effect of pretreating slices with 20 µM ketamine on synaptic plasticity in hippocampal slices from SD rats. **D** Time course of fEPSP slope normalized to baseline before LTP induction; where indicated, a subthreshold θ-burst stimulation was applied (*n* = 4–5 slices each). Representative traces shown on top. **E** Time course of normalized fEPSP slope measured in slices from SD and FSL rats. Where indicated, ketamine was applied to the FSL slices (*n* = 10–13 slices each). Representative traces shown on top. **F** Summary of normalized fEPSP slope measured 1 h after LTP induction under the indicated conditions *p < 0.05 versus DL-TBOA and §*p* < 0.05 versus the corresponding aCSF group (Student’s *t*-test). **G** Cartoon illustrating that homeostatic types of plasticity and activity induced potentiation act together to tune synaptic transmission, and that ketamine can act at several mechanisms to stabilize the tuning.

A previously suggested mechanism of action for ketamine is suppression of spontaneous NMDA receptor activation, inducing a rapid increase in evoked transmission via the BDNF-dependent recruitment of AMPA receptors [28]. We, therefore, tested whether ketamine can also affect the recovery phase of synaptic tuning measured 60 min after application of DL-TBOA under low-frequency (i.e., 0.001 Hz) stimulation (Fig. 5A, B). We found that the addition of either 20 µM ketamine or 10 ng/ml BDNF caused a significant recovery of fEPSP magnitude (*p* < 0.05 at 60 min compared to 75 min, ANOVA followed by Dunnett’s test in both conditions; Fig. 5A, B).

### Ketamine reverses the reduced threshold for synaptic plasticity

Finally, we examined whether briefly treating slices in the new steady state with ketamine could reverse the increased susceptibility to LTP induced with subthreshold θ-burst stimulation. We, therefore, treated SD slices prepared with DL-TBOA during 0.1-Hz stimulation to induce the new steady state, followed by treatment with ketamine for 30 min; after a wash-out period of 20 min, we then applied subthreshold θ-burst stimulation. We found that subthreshold stimulation failed to induce LTP in the ketamine-treated slices (*p* = 0.52 baseline versus 55–60 min after stimulation, student *t*-test; Fig. 5D, F). Moreover, treating slices obtained from FSL rats with ketamine also eliminated LTP induced by subthreshold stimulation (*p* = 0.72 baseline versus 55–60 min after stimulation, student *t*-test; Fig. 5E, F). Thus, brief application of a low dose of ketamine prevented all the retuning steps that we have observed in response to blocking glutamate uptake. The inducing theta burst simulation was not different in the ketamine pretreated slices compared to control, arguing against an incomplete wash-out of ketamine (Supplementary Fig. 6).

## Discussion

The ability to fine-tune synaptic transmission through interacting homeostatic and feed-forward plasticity mechanisms is the core of achieving a robust - yet highly plastic - system. Here, we report that increasing extracellular glutamate levels through blocking glutamate transporters induces several steps of changes in synaptic response during the first 1–1.5 h, that reminds of LTD as well as of homeostatic adaptation to inactivity. Rather than fitting these changes into any of the defined forms of plasticity we propose that synaptic strength is constantly changing and adapting, and that synaptic plasticity may be less categorical than what is typically described. We will therefore refer to the changes in synaptic strength that we observe as synaptic tuning. In support for the idea of overlapping forms of plasticity, we recorded both a quantitative change in synaptic strength and a change in the susceptibility to induce long-term potentiation in the same preparation. Thus, in contrast to the often-used metaphor of the synapse serving as a “volume control” in which neurotransmission can be dialed up or down, synaptic tuning may actually be more similar to a gearbox, in which the overall drive and acceleration are modulated (Fig. 5G).

A reduction of fEPSP induced by an increase in extrasynaptic glutamate levels have previously been described [29, 30], in agreement with our results, where direct recordings of extracellular glutamate with highly sensitive microelectrodes show that application of DL-TBOA increases baseline extracellular glutamate levels about 2-fold (Fig. 1B), reaching a level that activates mainly NMDA receptors [29]. In contrast, no difference in peak glutamate level was observed after stimulation in the presence of DL-TBOA, unlike what has been reported [24]. It is possible that the fast increase in peak glutamate level is exclusively dependent on release probability, and not affected by the rate of glutamate uptake. However, we cannot exclude that our method of detecting glutamate does not provide the spatial and temporal sensitivity for detecting changes in peak levels.

The overall time course of the retuning process, as well as its dependence on NMDA receptors, is similar to what is described for long-term depression (LTD) [31, 32] in contrast to homeostatic scaling where the downregulation is typically lower and slower [33]. Moreover, achieving a new steady-state is dependent on the retuning protocol, where high levels of glutamate induce a fast retuning that can be completely reversed after wash-out, in contrast to DL-TBOA application. Our interpretation is that several processes coexist that are constantly reshaping the synaptic strength, and that this process is more dynamic and state-dependent than previously thought. Interesting, in the visual cortex, LTD has been shown to be dependent of uncoherent input, [34] and an extended postsynaptic depolarization, which could be comparable to the effect of blocking glutamate transporters at the single cell level [35, 36]. Furthermore, the most prominent effect at the new steady-state that we describe is a reduced threshold for plasticity. An analogous result has previously been described, where reduced activity induced an increase in presynaptic activity and reduced probability to undergo synaptic potentiation [37]. A sliding threshold for plasticity has been extensively studied in the visual cortex in the so called BCM theory where prior synaptic activity and NMDA receptor activation reduces the threshold for plasticity [38, 39] similar to what we here describe in the hippocampus. Due to its relatively easy access, the visual cortex has served as a model for studying plasticity and adaptation in response to changes in activity [40], and our results suggest that reducing glutamate uptake in the hippocampus and monocular deprivation in the visual cortex use similar retuning mechanisms.

An intriguing finding in this work is that although we can record no synaptic response after 40 min of DL-TBOA, the subsequent recovery is dependent of presynaptic stimulation (Fig. 3). In the case of inactivity-induced homeostatic scaling, the underlying mechanism involves the spontaneous release of glutamate driving phosphorylation of eEF2 (eukaryotic elongation factor 2) and inhibition of local translation [41, 42]. Thus, in our case it is reasonable to speculate that presynaptic activity in the context of inhibited glutamate transporters relieves this effect on translation. The fact that ketamine could also counteract the lack of presynaptic activity and induced the recovery of fEPSPs, when applied 60 min after DL-TBOA treatment, is in good agreement with the previously suggested mechanism of action for ketamine: through the fast increase of BDNF via inhibition of postsynaptic NMDA receptors and dis-inhibition of protein translation [41, 42]. This mechanism is further supported by the fact that also exogenous administration of BDNF was able to rescue the recovery of fEPSPs in the absence of presynaptic activity.

The direct effect of reduced glutamate uptake on synaptic transmission is far from intuitive, and a variety of results have previously been described. An expected slowing of the decay of NMDA-, but not AMPA-mediated synaptic currents has been reported [43] in good agreement with our present work. A lack of effect on AMPA receptor mediated EPSCs amplitude and decay has previously been reported by us, together with an unexpected acute increase in frequency of events after DL-TBOA application [23]. Other studies have shown an acute reduction in fEPSP amplitude [29, 30], possibly explained by excessive activation of L-type channels [24]. One explanation for the variation in the results could be that the effect of reduced glutamate uptake is state-dependent, and that glutamate transporters are important to regulate the steady-state. The existence and robustness of a homeostatic “set point” of activity may be a fundamental feature of neuronal circuits achieved by a wide range of interacting mechanisms. Such an activity set point can ensure that brain activity is balanced despite perturbations, changes in activity levels over time, and even injury [44]. However, the price to pay for this stable system is that changes in the set point itself—as suggested in certain psychiatric disorders such as depression [1] and Alzheimer’s disease [45]—can be devastating. The set point of neuronal activity is regulated by synaptic plasticity/homeostasis [15], excitatory/inhibitory balance [46], and cellular excitability [45]. Here, we found that increasing extracellular glutamate by blocking glutamate transporters induces a long-lasting downregulation of the set point that remains even after the glutamate transporters are no longer blocked. Thus, blocking glutamate transporters induces a long-lasting change in steady-state synaptic activity and reduces the threshold for plasticity. Although, in this case, we change the set-point to a pathological state, the opposite manipulation could be tested for therapeutic effects. Moreover, the fact that the majority of glutamate transporters are expressed on astrocytes underscores the importance of these cells in maintaining balanced neuronal activity, as well as their potential as a therapeutic target for the treatment of certain psychiatric and cognitive disorders [47].

This study was designed to investigate the consequences of reduced glutamate transport on synaptic activity, given the pathogenic role that this reduction appears to play in a wide range of psychiatric disorders, including major depression [22]. Indeed, we found that the threshold for inducing LTP was significantly reduced in hippocampal slices prepared from FSL rats, an established animal model of depression with reduced expression of glutamate transporters [23]. Facilitated hippocampal LTP is not a feature classically associated with depression, in fact, we previously have reported that LTP amplitude is reduced in the FSL rat [23]. However, taken together, the reduced threshold and amplitude, could lead to an unstable network with increased randomness, a notion consistent with EEG recordings of patients with depression [48]. Interestingly, our results indicate that the NMDA receptor antagonist ketamine—used clinically as a fast-acting antidepressant—affects the “synaptic gearbox” at several sites. Not surprisingly, we found that ketamine prevents the decrease in synaptic strength induced by DL-TBOA, mimicking the effects of other NMDA receptor antagonists. Furthermore, ketamine was also effective when applied in the presence of DL-TBOA during low-frequency (0.001-Hz) stimulation, and ketamine prevented the increased susceptibility to LTP both in DL-TBOA‒treated slices obtained from wild-type rats and in slices obtained from FSL rats. Thus, ketamine appears to stabilize synaptic tuning regardless of the synapse’s previous state. A consequence of our reasoning is that previous treatment, stimulation protocol and baseline definition will affect how the effect of ketamine is registered. This may be the reason, why in our experiments, ketamine alone does not increase the synaptic response (Fig. 5) as have previously been convincingly shown [26, 42]. Moreover, it is interesting to note that structural plasticity was recently reported to be required for ketamine’s sustained effects, but not for the induction [12]. Thus, our finding of synaptic retuning suggests a potential mechanism underlying the rapid initial response to ketamine.

Taken together, these results support the emerging hypothesis that depression may be explained as a dysregulation of synaptic tuning rather than a change in excitation or inhibition per se. The interaction of different plastic mechanisms and the resulting synaptic retuning may therefore represent a promising new framework for developing new treatments for depression and related psychiatric disorders.

### Supplementary information

Supplementary Figures 1–6

## References

[1] Vose LR, Stanton PK (2017). Synaptic plasticity, metaplasticity and depression. Curr Neuropharmacol.

[2] Frere S, Slutsky I (2018). Alzheimer’s disease: from firing instability to homeostasis network collapse. Neuron.

[3] Page CE, Coutellier L (2019). Prefrontal excitatory/inhibitory balance in stress and emotional disorders: evidence for over-inhibition. Neurosci Biobehav Rev.

[4] Appelbaum LG, Shenasa MA, Stolz L, Daskalakis Z (2023). Synaptic plasticity and mental health: methods, challenges and opportunities. Neuropsychopharmacology.

[5] Berman RM, Cappiello A, Anand A, Oren DA, Heninger GR, Charney DS (2000). Antidepressant effects of ketamine in depressed patients. Biol Psychiatry.

[6] Zarate CA, Singh JB, Carlson PJ, Brutsche NE, Ameli R, Luckenbaugh DA (2006). A randomized trial of an N-methyl-D-aspartate antagonist in treatment-resistant major depression. Arch Gen Psychiatry.

[7] Duman RS, Sanacora G, Krystal JH (2019). Altered connectivity in depression: GABA and glutamate neurotransmitter deficits and reversal by novel treatments. Neuron.

[8] Wei Y, Chang L, Hashimoto K. Molecular mechanisms underlying the antidepressant actions of arketamine: beyond the NMDA receptor. Mol Psychiatry. 2021,27:559–73. 10.1038/s41380-021-01121-1 PMC8960399 33963284

[9] Kavalali ET, Monteggia LM (2020). Targeting homeostatic synaptic plasticity for treatment of mood disorders. Neuron.

[10] Abdallah CG, Sanacora G, Duman RS, Krystal JH (2015). Ketamine and rapid-acting antidepressants: a window into a new neurobiology for mood disorder therapeutics. Annu Rev Med.

[11] Cornwell BR, Salvadore G, Furey M, Marquardt CA, Brutsche NE, Grillon C (2012). Synaptic potentiation is critical for rapid antidepressant response to ketamine in treatment-resistant major depression. Biol Psychiatry.

[12] Moda-Sava RN, Murdock MH, Parekh PK, Fetcho RN, Huang BS, Huynh TN (2019). Sustained rescue of prefrontal circuit dysfunction by antidepressant-induced spine formation. Science.

[13] Zanos P, Gould TD (2018). Mechanisms of ketamine action as an antidepressant. Mol Psychiatry.

[14] Nicoll RA (2017). A brief history of long-term potentiation. Neuron.

[15] Turrigiano G (2012). Homeostatic synaptic plasticity: local and global mechanisms for stabilizing neuronal function. Cold Spring Harb Perspect Biol.

[16] Keck T, Toyoizumi T, Chen L, Doiron B, Feldman DE, Fox K (2017). Integrating Hebbian and homeostatic plasticity: the current state of the field and future research directions. Philos Trans R Soc B Biol Sci.

[17] Stampanoni Bassi M, Iezzi E, Gilio L, Centonze D, Buttari F (2019). Synaptic plasticity shapes brain connectivity: implications for network topology. Int J Mol Sci.

[18] Piva A, Caffino L, Mottarlini F, Pintori N, Castillo Díaz F, Fumagalli F (2021). Metaplastic effects of ketamine and MK-801 on glutamate receptors expression in rat medial prefrontal cortex and hippocampus. Mol Neurobiol.

[19] Thiagarajan TC, Lindskog M, Malgaroli A, Tsien RW (2007). LTP and adaptation to inactivity: overlapping mechanisms and implications for metaplasticity. Neuropharmacology.

[20] Murphy-Royal C, Dupuis J, Groc L, Oliet S (2017). Astroglial glutamate transporters in the brain: regulating neurotransmitter homeostasis and synaptic transmission. J Neurosci Res.

[21] Rajkowska G, Stockmeier CA (2013). Astrocyte pathology in major depressive disorder: insights from human postmortem brain tissue. Curr Drug Targets.

[22] Parkin GM, Udawela M, Gibbons A, Dean B (2018). Glutamate transporters, EAAT1 and EAAT2, are potentially important in the pathophysiology and treatment of schizophrenia and affective disorders. World J Psychiatry.

[23] Gómez-Galán M, De Bundel D, Van Eeckhaut A, Smolders I, Lindskog M (2013). Dysfunctional astrocytic regulation of glutamate transmission in a rat model of depression. Mol Psychiatry.

[24] Barnes JR, Mukherjee B, Rogers BC, Nafar F, Gosse M, Parsons MP (2020). The relationship between glutamate dynamics and activity-dependent synaptic plasticity. J Neurosci.

[25] Gómez-Galán M, Femenía T, Åberg E, Graae L, Van Eeckhaut A, Smolders I (2016). Running opposes the effects of social isolation on synaptic plasticity and transmission in a rat model of depression. PLoS One.

[26] Kim JW, Monteggia LM (2020). Increasing doses of ketamine curtail antidepressant responses and suppress associated synaptic signaling pathways. Behav Brain Res.

[27] Izumi Y, Zorumski CF (2014). Metaplastic effects of subanesthetic ketamine on CA1 hippocampal function. Neuropharmacology.

[28] Nosyreva E, Szabla K, Autry AE, Ryazanov AG, Monteggia LM, Kavalali ET (2013). Acute suppression of spontaneous neurotransmission drives synaptic potentiation. J Neurosci.

[29] Le Meur K, Galante M, Angulo MC, Audinat E (2007). Tonic activation of NMDA receptors by ambient glutamate of non-synaptic origin in the rat hippocampus. J Physiol.

[30] Potier B, Billard JM, Rivière S, Sinet PM, Denis I, Champeil-Potokar G (2010). Reduction in glutamate uptake is associated with extrasynaptic NMDA and metabotropic glutamate receptor activation at the hippocampal CA1 synapse of aged rats. Aging Cell.

[31] Collingridge GL, Peineau S, Howland JG, Wang YT (2010). Long-term depression in the CNS. Nat Rev Neurosci.

[32] Lee HK, Kameyama K, Huganir RL, Bear MF (1998). NMDA induces long-term synaptic depression and dephosphorylation of the GluR1 subunit of AMPA receptors in hippocampus. Neuron.

[33] Ibata K, Sun Q, Turrigiano GG (2008). Rapid synaptic scaling induced by changes in postsynaptic firing. Neuron.

[34] Linden ML, Heynen AJ, Haslinger RH, Bear MF (2009). Thalamic activity that drives visual cortical plasticity. Nat Neurosci.

[35] Henneberger C, Bard L, Panatier A, Reynolds JP, Kopach O, Medvedev NI (2020). LTP induction boosts glutamate spillover by driving withdrawal of perisynaptic astroglia. Neuron.

[36] Srivastava I, Vazquez-Juarez E, Lindskog M (2020). Reducing glutamate uptake in rat hippocampal slices enhances astrocytic membrane depolarization while down-regulating CA3–CA1 synaptic response. Front Synaptic Neurosci.

[37] Soares C, Lee KFH, Béïque J-C (2017). Metaplasticity at CA1 synapses by homeostatic control of presynaptic release dynamics. Cell Rep.

[38] Cooper LN, Bear MF (2012). The BCM theory of synapse modification at 30: interaction of theory with experiment. Nat Rev Neurosci.

[39] Rodriguez G, Mesik L, Gao M, Parkins S, Saha R, Lee HK (2019). Disruption of NMDAR function prevents normal experience-dependent homeostatic synaptic plasticity in mouse primary visual cortex. J Neurosci.

[40] Kaneko M, Stryker MP. Homeostatic plasticity mechanisms in mouse V1. Philos Trans R Soc Lond B Biol Sci. 2017;372:20160504. 10.1098/rstb.2016.0504 PMC5247599 28093561

[41] Sutton MA, Taylor AM, Ito HT, Pham A, Schuman EM (2007). Postsynaptic decoding of neural activity: eEF2 as a biochemical sensor coupling miniature synaptic transmission to local protein synthesis. Neuron.

[42] Autry AE, Adachi M, Nosyreva E, Na ES, Los MF, Cheng PF (2011). NMDA receptor blockade at rest triggers rapid behavioural antidepressant responses. Nature.

[43] Tsukada S, Iino M, Takayasu Y, Shimamoto K, Ozawa S (2005). Effects of a novel glutamate transporter blocker, (2S, 3S)-3-{3-[4-(trifluoromethyl)benzoylamino]benzyloxy}aspartate (TFB-TBOA), on activities of hippocampal neurons. Neuropharmacology.

[44] Ma Z, Turrigiano GG, Wessel R, Hengen KB (2019). Cortical circuit dynamics are homeostatically tuned to criticality in vivo. Neuron.

[45] Styr B, Slutsky I (2018). Imbalance between firing homeostasis and synaptic plasticity drives early-phase Alzheimer’s disease. Nat Neurosci.

[46] Field RE, D'amour JA, Tremblay R, Miehl C, Rudy B, Gjorgjieva J (2020). Heterosynaptic plasticity determines the set point for cortical excitatory-inhibitory balance. Neuron.

[47] Escartin C, Galea E, Lakatos A, O'Callaghan JP, Petzold GC, Serrano-Pozo A (2021). Reactive astrocyte nomenclature, definitions, and future directions. Nat Neurosci.

[48] Zhang M, Zhou H, Liu L, Feng L, Yang J, Wang G (2018). Randomized EEG functional brain networks in major depressive disorders with greater resilience and lower rich-club coefficient. Clin Neurophysiol.

