# Supplementary material for: The effect of ketamine on synaptic mistuning induced by impaired glutamate reuptake
Source: Neuropsychopharmacology. 2023 Jun 10;48(13):1859–68. doi: 10.1038/s41386-023-01617-0 (PMC10584870; doi:10.1038/s41386-023-01617-0)
Supplement: Supplementary file 1 — Supplementary Figures 1–6 [file 41386_2023_1617_MOESM1_ESM.pdf]

Supplementary Material

The effect of a subanesthetic dose of ketamine on synaptic mistuning induced by impaired glutamate reuptake

Authors: Erika Vazquez-Juarez, PhD[$]; Ipsit Srivastava, PhD[‡;] Maria Lindskog, PhD[§]

Supplementary Figures 1-6

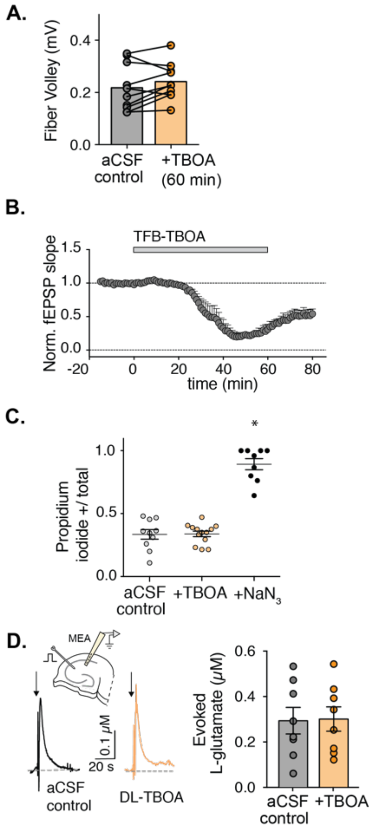

Supplementary figure 1.

Fig. S1. A) Action potentials induced by the stimulation was quantified as the amplitude of the fiber volley in a subset of recordings. DL-TBOA did not affect fiber volley amplitude, indicating that the presynaptic input was not affected by blocking of glutamate transporters. B) TFB-TBOA, a glutamate transporter blocker with higher specificity for isoforms expressed mainly in astrocytes, evoked the same re-tuning of the synapse as DL-TBOA. fEPSP slope over time plotted as average of 6 stimulation pulses per minute (concentration of TFB-TBOA: 200 nM; n=4). C) The effect of 60 min exposure to DL-TBOA (n=13), aCSF alone (n=10) or sodium azide (1mM, n=9) on cell viability determined by propidium iodine staining and presented as fraction of total number of cells that have taken up propidium iodine. D) DL-TBOA did not affect peak glutamate levels after synaptic activation, as stimulation of the Schaffer collaterals increased extracellular glutamate to the same level (approximately 300 nM) regardless of the presence or absence of DL-TBOA. Example traces (left) of extracellular glutamate recordings during Schaffer collateral stimulation (arrows) and average peak glutamate concentration (right); n=7 and 8 slices for control and 50 µM DL-TBOA, respectively.

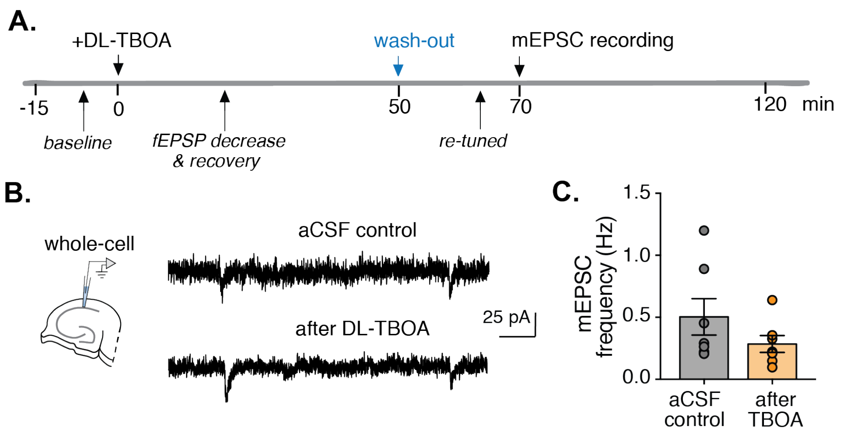

Supplementary figure 2.

Fig. S2. We recorded miniature excitatory postsynaptic currents (mEPSCs) in slices in which the synapses had been retuned with DL-TBOA and in control slices in which DL-TBOA was replaced with aCSF. A) Protocol used to induce a new steady state in hippocampal slices by applying 50 μM DL-TBOA for 40 minutes while stimulating the Schaffer collaterals at 0.1 Hz. Control slices are treated similarly but are perfused with aCSF instead of DL-TBOA. B) Representative traces of patch-clamp recordings in control and retuned slices; tetrodotoxin was present throughout the recordings to isolate mEPSCs. C) Summary of the frequency of mEPSCs recorded in control slices and retuned slices (n=7 slices each) show no significance difference between the two conditions.

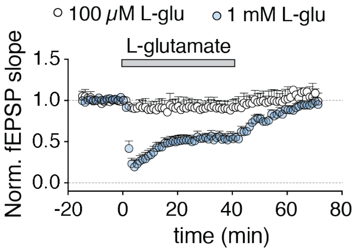

Supplementary figure 3 .

Fig. S3. We hypothesize that the neuronal network can initially compensate for the effects of an increase in extracellular glutamate, but this compensatory mechanism is likely overwhelmed when extracellular glutamate is chronically increased. Consistent with this suggested resilience of the system, bath application of a relatively low concentration of glutamate (100 μM) had no significant effect on fEPSPs, whereas application of 1 mM glutamate induced a change in fEPSPs that was qualitatively similar to the effects observed with DL-TBOA.

Time-course of fEPSP at application of 1 mM or 100 μM L-glutamate. Glutamate was applied where indicated (n=5 slices each).

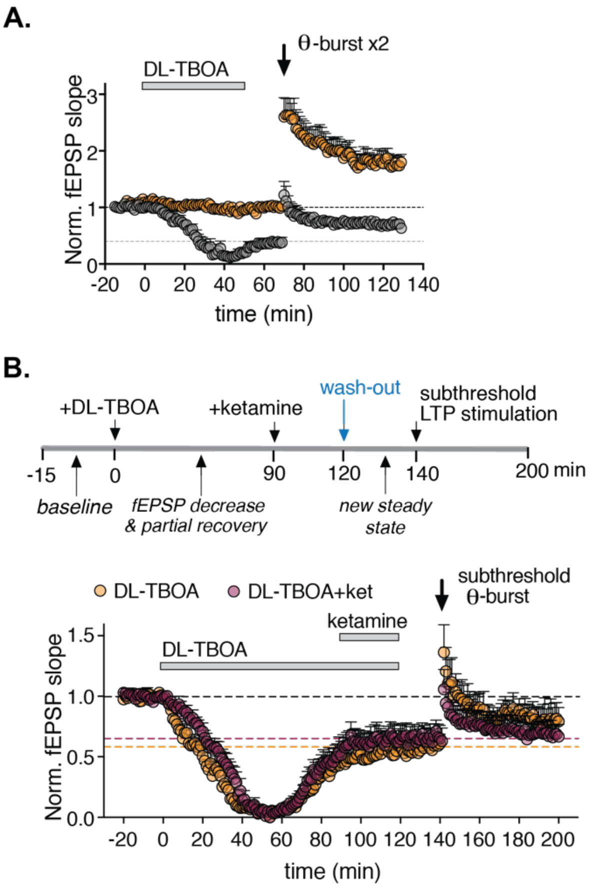

Supplementary figure 4.

Fig S4. A) fEPSP over time normalized to initial fEPSP baseline. 20 minutes after DL-TBOA wash-out, at the new steady state, Schaffer collaterals were stimulated with a standard LTP inducing theta burst applied at 60 minutes, n=5 for control, 4 for DL-TBOA treated. B) fEPSP over time normalized to initial fEPSP baseline, 90 minutes after DL-TBOA application, n=5 for ketamine + DL-TBOA treated slices, n=11 for DL-TBOA treated slices. Error bars denote standard error of the mean (SEM).

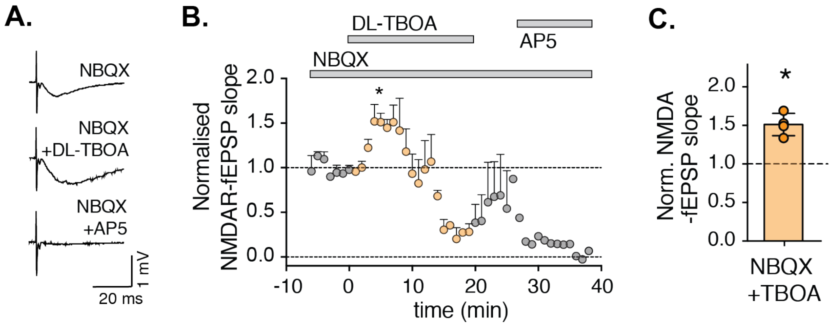

Supplementary figure 5.

Fig. S5. A) NMDA receptor–mediated field potentials recorded in the presence of 25 µM NBQX and 0.2 mM $MgCl_2$; bath application of 50 µM AP5 eliminated the response (bottom trace). B) NMDA receptor-mediated component of the fEPSP over time (25 µM NBQX in 0.2 mM MgCl2 and fully inhibited by AP5) at application of 50 µM DL-TBOA and its'wash-out. C) Average fEPSP slope measured 5 min after application of NBQX + DL-TBOA, normalized to NBQX alone (n=4 slices each). *p<0.05 (Mann-Whitney U test).

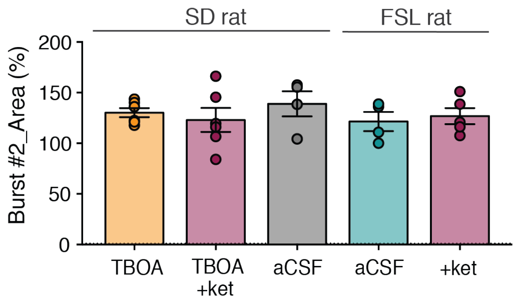

Supplementary figure 6.

Fig. S6 Previous exposure to DL-TBOA did not affect the intensity of the theta burst stimulation used to induce LTP. Theta burst intensity for DL-TBOA treated as well as FSL groups was quantified as area of the recorded burst (amplitude x time) and no significant difference between groups was observed (One-way ANOVA; n=4-6). Average area for each experimental group shown, error bars denote standard error of the mean (SEM).
